# A Novel Model Based on Serum Biomarkers to Predict Primary Non-Response to Infliximab in Crohn’s Disease

**DOI:** 10.3389/fimmu.2021.646673

**Published:** 2021-07-22

**Authors:** Li Li, Rirong Chen, Yingfan Zhang, Gaoshi Zhou, Baili Chen, Zhirong Zeng, Minhu Chen, Shenghong Zhang

**Affiliations:** Division of Gastroenterology, The First Affiliated Hospital, Sun Yat-sen University, Guangzhou, China

**Keywords:** Crohn’s disease, primary non-response, infliximab, serum biomarkers, prediction

## Abstract

**Background:**

Infliximab is effective in inducing and maintaining remission in patients with Crohn’s disease (CD), but primary non-response (PNR) occurs in 10-30% of cases. We investigated whether serum biomarkers are effective in predicting PNR in patients with CD.

**Methods:**

From January 2016 to April 2020, a total of 260 patients were recruited to this prospective and retrospective cohort study. Serum samples were collected at baseline and week 2 of infliximab treatment. Serum levels of 35 cytokines were assessed in 18 patients from the discovery cohort and were further evaluated in the 60-patient cohort 1. Then, candidate cytokines and other serological biomarkers were used to construct a predictive model by logistic regression in a 182-patient cohort 2. PNR was defined based on the change of CD activity index or clinical symptoms.

**Results:**

Among the 35 cytokines, matrix metalloproteinase 3(MMP3) and C-C motif ligand 2 (CCL2) were two effective serum biomarkers associated with PNR in both the discovery cohort and cohort 1. In cohort 2, serum level of MMP3, CCL2 and C-reactive protein (CRP) at 2 weeks after infliximab injection were independent predictors of PNR, with odds ratios (95% confidence interval) of 1.108(1.059-1.159), 0.940(0.920-0.965) and 1.102(1.031-1.117), respectively. A PNR classifier combining these three indicators had a large area under the curve [0.896(95% CI:0.895-0.897)] and negative predictive value [0.918(95%CI:0.917-0.919)] to predict PNR to infliximab.

**Conclusions:**

MMP3, CCL2, and CRP are promising biomarkers in prediction of PNR to infliximab, and PNR classifier could accurately predict PNR and may be useful in clinical practice for therapy selection.

## Introduction

The introduction of infliximab (IFX), a chimeric monoclonal antibody against tumour necrosis factor (TNF)-α, has significantly improved therapy to induce and maintain remission in Crohn’s disease (CD) (1). However, 10-30% of patients receiving IFX are non-responsive during induction therapy (primary non-response, PNR) (2). Furthermore, IFX therapy is expensive and may give rise to some adverse events, such as infusion reactions and infections (1). Incorrect use of IFX in PNR patients would delay treatment as well as lead to disease progression (3). Thus, it is important to explore a method for precisely predicting PNR in patients of CD.

Previous studies have demonstrated that clinical characteristics (4, 5), including disease duration, age at diagnosis, concomitant use of immunosuppressant drugs, and disease location or behaviour, were associated with the efficacy of IFX. Besides, genetic polymorphisms (6, 7), microbiome (8) and serum biomarkers (5, 9) have also been studied with regard to response to IFX. Although some risk factors of PNR were confirmed, the mechanisms underlying PNR have not been broadly defined. It has been widely proposed that non-TNF-α mediated inflammation may result in PNR to IFX and some pro-inflammatory pathways could even be regulated by TNFα blockade (10). Therefore, we believe that the inflammatory state of patients could affect the IFX response, and alterations of inflammatory cytokines in serum might be an appropriate biomarker to predict PNR.

Previous studies have shown that inflammatory cytokines, including TNF-α, interleukin (IL)-6, IL-1β, IL-17A, IL-23, and IL-12, may be predictive factors of PNR to IFX (11–13). However, these studies only found the discrepant expression levels of the cytokines between the respondent and non-respondent groups, and none of these cytokines has been widely used in clinical practise. Thus, identifying effective serum cytokines that affect therapeutic failure could help select the most appropriate patients for IFX treatment. In this prospective and retrospective cohort study, we assessed circulating inflammatory cytokines levels before and after the administration of IFX to identify potential serum biomarkers, and then constructed a model in prediction of PNR to IFX in CD patients.

## Patients and Methods

### Study Design and Patient Population

This was a prospective and retrospective, single-centre cohort study, approved by the institutional ethics committee (IEC) for Clinical Research and Animal Trials of the First Affiliated Hospital of Sun Yat-sen University (No. 2019-383). Informed consent was obtained from all patients. Patients with a definite diagnosis of CD and receiving IFX therapy in the First Affiliated Hospital of Sun Yat-sen University were consecutively included in the study from January 2016 to April 2020. Other inclusion criteria were as follows: (1) treatment with 5 mg/kg IFX intravenously at week 0, 2, and 6; (2) naïve to anti-TNF and other biologicals therapy; and (3) CD activity index (CDAI) of ≥150 before IFX treatment. Exclusion criteria included the following: (1) lack of evaluation for disease activity at baseline or at week 14 after the first treatment with IFX; (2) unavailable blood samples at baseline and week 2; (3) delayed infusion; (4) pregnancy; and (5) lost to follow-up.

The Montreal system was used to classify the location and behaviour of CD patients (14). The CDAI was calculated for all patients at baseline and week 14 after IFX therapy. At baseline, clinical characteristics including sex, age at first IFX therapy, age at diagnosis, disease duration, body mass index, disease location and behaviour, presence of extraintestinal manifestations, history of smoking and surgery, concomitant azathioprine, and CDAI score were recorded. The endoscopic activity of patients was assessed using the Simple Endoscopic Score for Crohn’s Disease (SESCD). C-reactive protein (CRP), erythrocyte sedimentation rate (ESR), albumin, haemoglobin, white blood cell, neutrophil, lymphocytes, and platelets counts were assessed at baseline and week 2 after IFX therapy.

PNR of IFX was defined as a failure of the CDAI to drop more than 70 points or to <150 at week 14 after IFX administration. Situations in which patients received an alternative therapy schedule such as an escalation of corticosteroid therapy, switching to other agents or having the surgery before week 14 were also defined as PNR.

**Figure 1:**
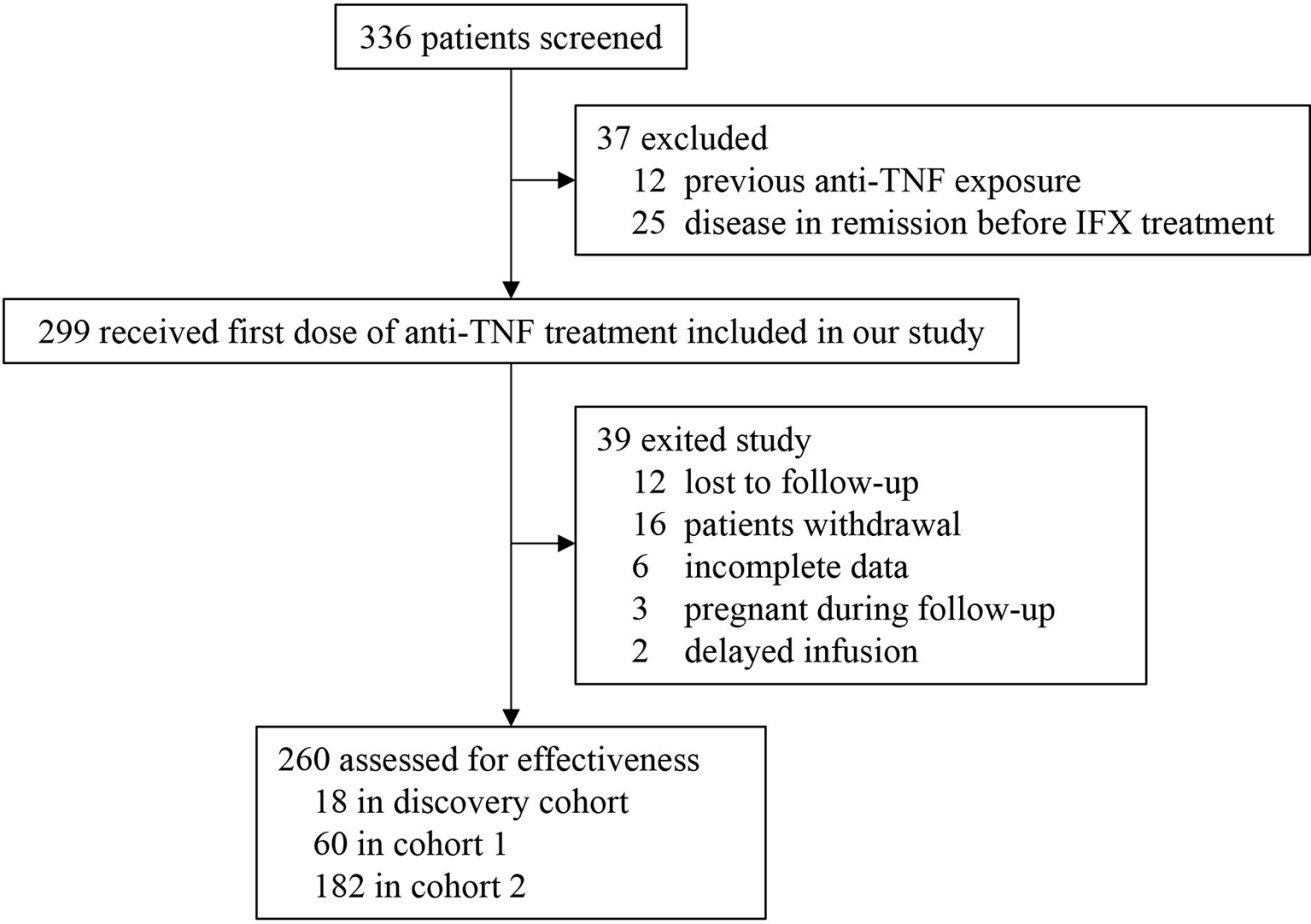
The flow chart of patient recruitment.

In this prospective and retrospective study, a total of 260 patients were eligible (**Figure 1**). Retrospective single-centre cohorts were designed to screen (discovery cohort, n=18) and validate (Cohort 1, n=60) the candidate cytokines. The first 9 primary responders and 9 non-responders constituted a discovery cohort to identify potential biomarkers associated with PNR. Serum levels of 35 cytokines at baseline and week 2 after IFX therapy were examined by the Luminex cytokine multiplex assay in 18 patients from the discovery cohort. The latter 60 patients were recruited into cohort 1 to further verify and screen the candidate cytokines selected from the discovery cohort. Thereafter, a prospective single-centre cohort (cohort 2, n=182) was recruited from May 2018 to April 2020 in the First Affiliated Hospital of Sun Yat-sen University, which were used to construct a predictive model based on the findings from cohort 1 and do internal validation through the bootstrap validation approach.

### Sample Collection

The blood samples were collected into serum separator tubes (BD Biosciences, Franklin Lakes, NJ, USA) before intravenous injection of Infliximab at baseline and week 2. The tubes were centrifuged at 1900× g for 15 minutes, then the serum samples were aliquoted with 0.5ml tubes (Ambion, Foster City, CA) and stored at −80°C until analyses.

### Assay for Serum Biomarkers

Serum levels of 35 cytokines, including C-C motif ligand (CCL)2, CCL4, CCL11, CCL20, CCL25, CCL26, CX3CL1, CXCL8, CXCL11, ADAMTS13, adiponectin, α-2-macroglobulin, matrix metalloproteinase (MMP)3, insulin-like growth factor binding protein-1, TNF-α, IL-1ß, IL-2, IL-4, IL-6, IL-7, IL-10, IL-11, IL-12 p70, IL-13, IL-17, IL-18, IL-23, IL-27, IL-28A, IL-28B, IL-31, IL-33, IL-34, and IL-36ß, at baseline and week 2 in discovery cohort were measured using a Luminex cytokine assay kit (LXSAHM-35) and a Luminex X-200 instrument according to the manufacturer’s protocol (R&D Systems, Minneapolis, MN, USA).

Enzyme-linked immunosorbent assay (ELISA) was used to measure levels of the molecules selected after the analysis of Luminex results obtained in the discovery cohort. In cohort 1 and 2, serum MMP3 levels were measured using a quantitative sandwich enzyme immunoassay (R&D Systems) at baseline and week 2. The serum CCL2 concentration was measured at week 2 using an ELISA kit (Abcam, Cambridge, UK, Ab179886). Serum IL-7 levels were measured using an ELISA kit (R&D Systems). Other indicators, such as CRP, ESR, albumin and so on, were tested with standard institutional protocols by the laboratory physicians at the First Affiliated Hospital of Sun Yat-sen University.

### Statistical Analyses

Continuous and categorical variables were described as medians with interquartile ranges and frequency with percentages, respectively. The non-parametric Wilcoxon and chi-square tests for continuous and categorical variables, respectively, were used to assess differences between responders and non-responders. The False Discovery Rate (Benjaminiand-Hochberg method) was performed to adjust the p-value in the comparison of 35 serum biomarkers between primary responders and non-responders in the discovery cohort. A p-value of <0.05 denoted statistical significance. Multivariate logistic regression (enter) was used to evaluate the relationship between the clinical or serological variables with the significant differences in the univariate analysis and the outcome of PNR among all patients in cohort 2, and also in patients with determined response status by CDAI in cohort 2. The variables with a p-value of <0.05 in the multivariate analyses were defined as independent predictive indicators, and were selected to construct a logistic regression model, the PNR classifier. Multivariate logistic regression was also performed to calculate the odds ratio (OR) of the independent predictive indicators and PNR classifier in predicting PNR. The area under the receiver operating characteristics curve (AUC), sensitivity, specificity, positive predictive value and negative predictive value were used to assess the predictive ability of the model, and their respective mean [95% confidence interval (CI)] were calculated by bootstrapping with 2,000 replications. Moreover, the bootstrap validation approach was also used to assess the internal validation of the independent predictive indicators and the PNR classifier for predicting PNR in cohort 2. R software, version 4.0.0, was used for statistical analyses.

## Results

### Patients Characteristics

Between January 2016 and April 2020, 260 patients were included in this prospective and retrospective cohort study (**Figure 1**). Baseline characteristics for discovery cohort, and cohorts 1 and 2 are documented in **Supplement Table 1** and **Table 1**, respectively. Only perianal disease (p<0.001) and serum albumin concentration (p=0.019) were significantly different between the two cohorts. In PNR patients, only ESR (p=0.015) was significantly different between cohort 1 and cohort 2 (**Supplement Table 2**). To evaluate the disease activity at week 14 more objectively, we performed a comparison of CRP, ESR and SESCD between primary responders and primary non-responders (**Supplement Table 3**). The results showed that primary non-responders had higher CRP, ESR and SESCD levels than primary responders at week 14 in both cohort 1 (CRP: p<0.001; ESR: p<0.001; SESCD: p<0.001) and cohort 2 (CRP: p<0.001; ESR: p<0.001; SESCD: p<0.001).

**Table 1:** Baseline characteristics of Cohort 1 and 2.

	Cohort 1 (n=60)		Cohort 2 (n=182)		P value*
	All patients	PNR patients	All patients	PNR patients	
Primary non-responders	12 (20)	12 (100)	45 (24.7)	45 (100)	0.489
Failure of CDAI reduction		10 (83.3)		38 (84.4)	
Therapy alteration		2 (16.7)		7 (15.6)	
Male	47 (78.3)	9 (75.0)	128 (70.3)	32 (71.1)	0.249
Age at 1^st^ IFX therapy (years)	24.5 (18.5-29.0)	24.38 (19.2-34.7)	24.9 (18.0-34.0)	28.8 (22.6-36.5)	0.242
Body-mass index (kg/m²)	17.1 (15.8-19.4)	17.7 (16.0-21.3)	17.7 (16.1-19.2)	17.4 (15.8-18.8)	0.371
Age at diagnosis (years)	21.8 (17.4-26.2)	22.6 (18.3-27.0)	22.0 (16.7-29.0)	23.0 (18.3-29.3)	0.775
Disease duration (years)	1.0 (0.5-3.9)	1.3 (0.8-8.4)	1.6 (0.7-4.9)	4.6 (1.0-6.9)	0.100
Disease location					0.373
L1 (ileal disease)	6 (10.0)	2 (16.7)	30 (16.5)	11 (24.4)	
L2 (colonic disease)	4 (6.7)	0 (0)	8 (4.4)	1 (2.2)	
L3 (ileocolonic disease)	50 (83.3)	10 (83.8)	144 (79.1)	33 (73.3)	
Presence of upper GI disease	11 (18.3)	2 (16.7)	42 (23.1)	9 (20.2)	0.478
Disease behavior					0.382
B1 (non stricturing, non-penetrating)	37 (61.7)	6 (50.0)	95 (52.2)	13 (28.9)	
B2 (stricturing)	18 (30.0)	4 (33.3)	62 (34.1)	25 (55.6)	
B3 (penetrating)	5 (8.3)	2 (16.7)	25 (13.7)	7 (15.6)	
Perianal disease	16 (26.7)	4 (33.3)	70 (38.5)	14 (31.1)	<0.001
Presence of extraintestinal manifestations	6 (10)	2 (16.7)	34 (18.7)	11 (24.4)	0.160
Previous surgery	8 (13.3)	2 (16.7)	40 (22.0)	13 (28.9)	0.191
History of smoking	3 (5)	0 (0)	15 (8.2)	3 (6.7)	0.573
Concomitant Azathioprine	32 (53.3)	8 (66.7)	79 (43.4)	19 (42.2)	0.181
CDAI score	251 (199-296)	268 (194-296)	234 (198-282)	232 (198-245)	0.519
C-reactive protein (mg/L)	29.4 (9.7-49.0)	33.0 (19.3-58.6)	21.7 (8.5-39.5)	19.4 (2.3-35.0)	0.154
Erythrocyte sedimentation rate (mm/h)	52.0 (31.3-70.0)	69.0 (47.5-85.8)	42.0 (24.0-72.0)	38.0 (19.5-60.5)	0.253
Albumin (g/L)	32.9 (30.0-36.3)	34.0 (30.8-36.5)	35.0 (31.2-38.9)	35.4 (31.2-38.2)	0.019
Haemoglobin (g/L)	111 (92-124)	113 (100-127)	113 (96-128)	114 (95-135)	0.319
Platelet count (×10^9^/L)	348 (259-461)	364 (301-442)	350 (280-450)	343 (267-459)	0.910

*The p value was from the non-parametric Wilcoxon test or chi-square test in comparison of baseline characteristics between all patients in cohort 1 and cohort 2.

Continuous variables and categorical variables are described as median (IQR) and n (%), respectively.

IQR, interquartile range; GI, gastrointestinal; CDAI, Crohn’s disease activity index.

### Distinct Serum Cytokine Profiles in CD Patients Receiving IFX Treatment

In the discovery cohort, Luminex multiplex assay was performed using serum samples collected before IFX treatment and at week 2. Through non-parametric Wilcoxon test, we found that the serum CCL2 level at week 2 (p=0.013), MMP3 level at baseline (p=0.050), MMP3 level at week 2 (p=0.008), and the change of IL-7 from baseline to week 2 (ΔIL-7) (p=0.050) were significantly different between the primary responders and non-responders (**Supplement Table 4**). However, after adjusting the p-value by False Discovery Rate, none of the indicators was significantly different.

**Figure 2:**
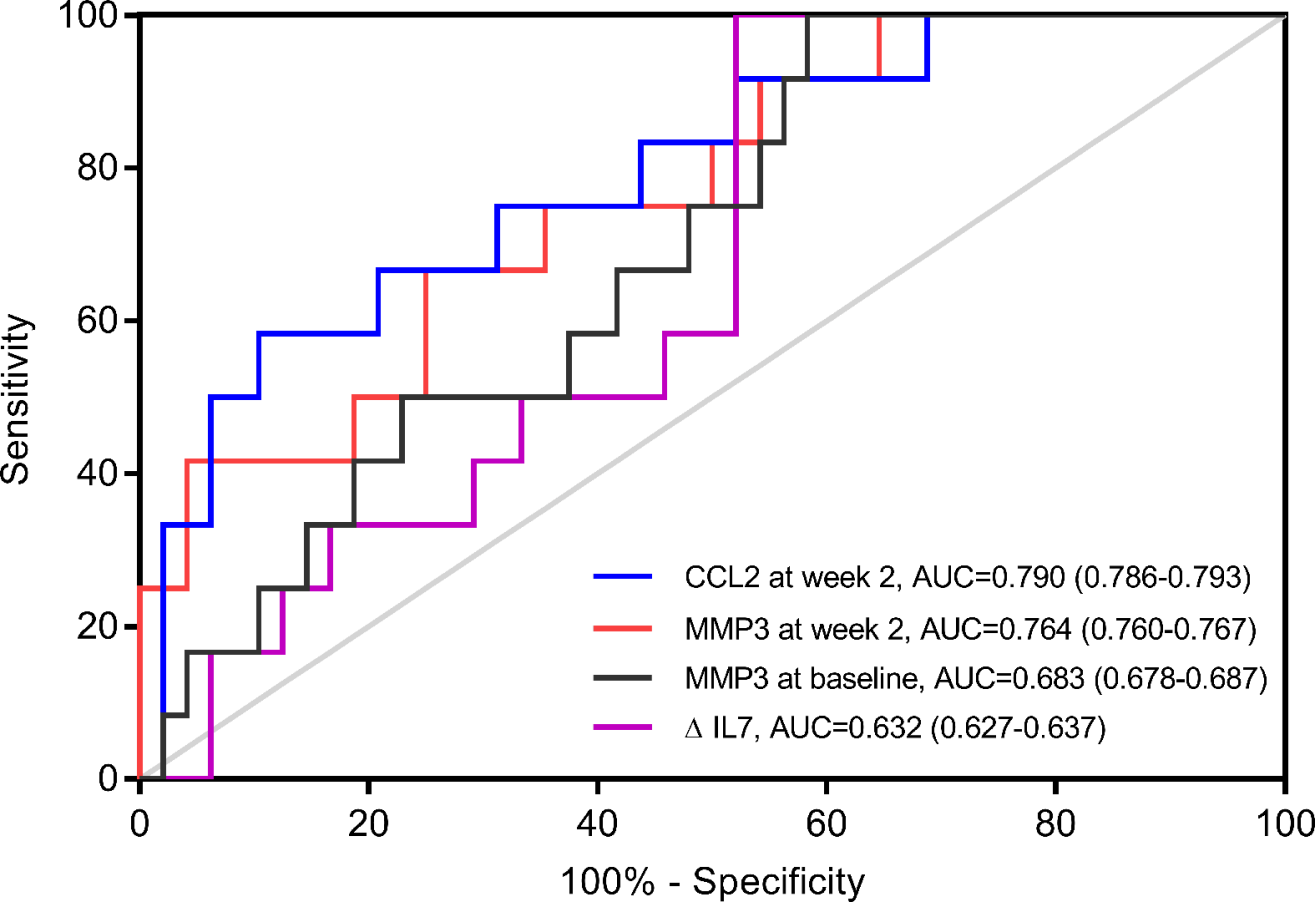
Receiver operating characteristics (ROC) analyses of potential predictors in cohort 1. ROC Curve of change of IL-7 from baseline to week 2 (ΔIL-7), matrix metalloproteinase (MMP) 3 at baseline, MMP3 at week 2 and C-C motif ligand (CCL) 2 at week 2 for predicting primary non-response (n=12) in cohort 1 (n=60).

To verify the discrepant levels of these four serum indicators, we performed further analyses by ELISA in cohort 1. MMP3 level at baseline (p=0.040), MMP3 level at week 2 (p=0.004) and CCL2 level at week 2 (p=0.001) showed significant difference between primary non-responders and responders, while ΔIL-7 showed no significant change (p=0.094) (**Table 2**). Furthermore, we observed that CCL2 and MMP3 levels at week 2 were significantly altered in the multivariate analysis; the odds ratios (ORs) (95% CI) were 0.903 (0.877-0.986, p=0.010) and 1.203 (1.039-1.394, p=0.008), respectively. The receiver operating characteristics analysis revealed that CCL2 [AUC (95% CI): 0.790 (0.786-0.793)] and MMP3 [AUC (95% CI): 0.764 (0.760-0.767)] levels at week 2 could better predict PNR than MMP3 at baseline [AUC (95% CI): 0.683 (0.678-0.687)] and ΔIL-7 [AUC (95% CI): 0.632 (0.627-0.637)] (**Figure 2**).

**Table 2:** Comparison of serum biomarkers detected by ELISA between primary non-responders and responders in cohort 1.

	Univariate analysis			Multivariate analysis	
	Primary response (n=48)	PNR (n=12)	P value	Odds Ratio (95% CI)	P value
ΔIL-7 (pg/ml)	-5.5 (-10.1- -3.2)	-4.5 (-6.0- -2.1)	0.095	1.261 (0.948-1.677)	0.111
MMP3 at baseline (ng/mL)	21.4 (13.6-27.0)	26.4 (21.2-35.7)	0.040	0.943 (0.869-1.024)	0.161
MMP3 at week 2 (ng/ml)	13.5 (8.2-18.5)	19.7 (14.2-38.3)	0.004	1.203 (1.039-1.394)	0.013
CCL2 at week 2 (pg/ml)	93.7 (77.3-128.5)	63.6 (59.3-86.7)	0.001	0.903 (0.877-0.986)	0.015

ELISA, Enzyme-linked immunosorbent assay; ΔIL-7, change of IL-7 from baseline to week 2; CCL, C-C motif ligand; MMP, matrix metalloproteinase.

### CCL2, MMP3, and CRP at Week 2 Are Independently Predictive of PNR

Considering that CCL2 and MMP3 at week 2 were the most important predictors of PNR to IFX, we further analysed these two indicators, as well as other clinical and serological markers, in cohort 2.

Univariate analyses showed that three clinical characteristics and eight serological indicators, including age at first IFX therapy (p=0.044), disease duration (p=0.002), CD behaviour (p=0.001), neutrophil percentage (p=0.010), lymphocytes counts(p=0.042), lymphocyte percentage (p=0.007), platelet-to-lymphocyte ratio (p=0.028), neutrophil-to-lymphocyte ratio (p=0.008), CRP (p<0.001), MMP3 (p<0.001) and CCL2 levels (p<0.001) at week 2, were significantly different between primary non-responders and responders (**Table 3** and **Supplemental Table 5**).

**Table 3:** Significant univariate association of clinical characteristics and serological markers for primary non-response in cohort 2.

	Univariate analysis			Multivariate analysis	
	Primary response (n=137)	PNR (n=45)	P value	Odds Ratio (95% CI)	P value
**Clinical characteristics**					
Age at 1^st^ IFX therapy (years)	23.6 (18.3-32.6)	28.8 (22.6-36.5)	0.044	1.012 (0.953-1.076)	0.692
Disease duration (years)	1.2 (0.6-4.0)	4.6 (1.0-6.9)	0.002	1.069 (0.964-1.185)	0.205
CD behaviour			0.001	1.540 (0.765-3.099)	0.226
B1 (non stricturing, non-penetrating)	82 (59.9)	13 (28.9)			
B2 (stricturing)	37 (27.0)	25 (55.6)			
B3 (penetrating)	18 (13.1)	7 (15.6)			
**Level of serological markers** **at weeks 2**					
Neutrophil percentage (%)	58.6 (51.8-64.1)	63.6 (55.8-71.7)	0.010	1.010 (0.902-1.131)	0.866
Lymphocytes (×10^9^/L)	1.6 (1.3-2.0)	1.4 (1.1-1.9)	0.042	1.658 (0.468-6.064)	0.425
Lymphocyte percentage (%)	30.6 (24.5-35.1)	24.9 (17.8-32.2)	0.007	0.974 (0.838-1.132)	0.727
PLR	180 (142-230)	217 (152-291)	0.028	1.005 (0.997-1.013)	0.243
NLR	2.0 (1.5-2.6)	2.5 (1.7-3.8)	0.008	0.980 (0.301-3.191)	0.947
CRP (mg/L)	1.5 (0.8-3.6)	4.0 (1.0-8.7)	<0.001	1.102 (1.031-1.117)	0.004
CCL2 (pg/ml)	98.9 (80.0-120.5)	72.0 (55.7-88.0)	<0.001	0.940 (0.920-0.965)	<0.001
MMP3 (ng/ml)	12.7 (8.1-17.4)	24.7 (16.0-33.6)	<0.001	1.108 (1.059-1.159)	<0.001

PLR, platelet-to-lymphocyte ratio; NLR, neutrophil-to-lymphocyte ratio; CRP, C-reactive protein; CCL, C-C motif ligand; MMP, matrix metalloproteinase.

Multivariate analyses of all patients in cohort 2 revealed that only CRP [OR 1.102 (95%CI: 1.031-1.117), p=0.004], MMP3 [OR 1.108 (95%CI: 1.059-1.159), p<0.001], and CCL2 levels at week 2 [OR 0.940 (95%CI: 0.920-0.965), p<0.001] were independently associated with PNR (**Table 3**). Another multivariate analysis was performed in patients with determined response status by CDAI, and the result was similar to that obtained while including all patients (**Supplement Table 6**).

### A Novel Model for Predicting PNR

The predictive values of CRP, CCL2, and MMP3 at week 2 are shown in **Table 4**. Both CRP and MMP3 had large specificity [(95% CI): 0.768 (0.765-0.770) and 0.799 (0.796-0.802), respectively], whereas CCL2 had high sensitivity [0.783 (0.778-0.788)], but low specificity [0.687 (0.681-0.692)].

**Table 4:** The ability of PNR classifier in predicting primary non-response to IFX.

	AUC (95% CI)	Cut-off value (95% CI)	Sensitivity (95% CI)	Specificity (95% CI)	PPV (95% CI)	NPV (95% CI)
PNR classifier	0.896 (0.895-0.897)	0.331 (0.327-0.335)	0.758 (0.755-0.762)	0.888 (0.885-0.890)	0.703 (0.699-0.708)	0.918 (0.917-0.919)
CRP at week 2	0.679 (0.677-0.681)	3.79 (3.76-3.82)	0.614 (0.610-0.617)	0.768 (0.765-0.770)	0.468 (0.465-0.471)	0.859 (0.857-0.860)
CCL2 at week 2	0.781 (0.779-0.782)	84.84 (84.44-85.23)	0.783 (0.778-0.788)	0.687 (0.681-0.692)	0.468 (0.465-0.472)	0.912 (0.910-0.913)
MMP3 at week 2	0.779 (0.777-0.781)	19.15 (19.05-19.25)	0.712 (0.709-0.716)	0.799 (0.796-0.802)	0.548 (0.544-0.551)	0.895 (0.894-0.897)

PPV, Positive predictive value; NPV, Negative predictive value; PNR, Primary non-response; PLR, platelet-to-lymphocyte ratio; CRP, C-reactive protein; CCL, C-C motif ligand; MMP, matrix metalloproteinase.

To better predict PNR, we built a PNR classifier combining serum levels of CRP, CCL2, and MMP3 at week 2 through logistic regression, and the variance inflation factors of CRP, MMP3 and CCL2 was equal to 1.004, 1.008 and 1.012, respectively. PNR classifier had a large AUC of 0.896 (95% CI: 0.895-0.897) to predict PNR (**Table 4**). PNR classifier also had the largest specificity [0.888 (95%CI: 0.885-0.890)], negative predictive value [0.918 (95%CI: 0.917-0.919)] and positive predictive value [0.703 (95%CI: 0.699-0.708)] in the prediction of PNR among these 4 indicators. The OR for getting PNR in patients with a value of PNR classifier > 0.331 was 6.467 (95% CI: 1.928-21.687; p=0.002), after adjustment for covariates (**Supplement Table 7**). Furthermore, patients with serum concentration of MMP3 > 19.15pg/ml [OR (95% CI): 8.478 (2.307-31.160); p=0.001] or CRP > 3.79 mg/L [OR (95% CI): 6.314 (1.956-20.377); p=0.002] also had the high risk to getting PNR.

## Discussion

IFX therapy is currently an important choice to induce and maintain remission in CD patients, but approximately 30% of patients exhibit PNR to IFX (1, 2). To achieve the goal of treating each patient at the right time with the optimal drug, it is necessary to detect which group of patients are unlikely to benefit from IFX treatment. In this prospective and retrospective cohort study of 260 CD patients, we found that clinical characteristics such as older age at first IFX therapy, longer disease duration, and stricturing behaviour were significantly associated with PNR to IFX. Furthermore, serum CRP, MMP3, and CCL2 levels at week 2 after IFX injection were the three independent predictors for PNR. Additionally, to better monitor CD, we further established a PNR classifier incorporating these three inflammation mediators, which was demonstrated to have a larger AUC (0.898 [95% CI: 0.837-0.947]) in discriminating primary non-responders from primary responders than using CRP, MMP3 or CCL2 independently. Thus, it can effectively identify patients who are suitable for IFX induction therapy.

MMP3 is a pattern of the large family of zinc dependent matrix metalloproteinases (MMPs), which plays an active role in the pathogenesis of inflammation in inflammatory bowel disease (IBD). The expression and secretion of MMPs are sensitive to the condition of intestinal inflammation, and are either augmented or moderated by a series of inflammatory cytokines, especially TNF-α (15). MMP3 is regarded as a crucial effector molecule of inflammatory cells, while it can also modify cytokines and chemokines (15). Thus, the up-regulated expression of MMPs prompts an initiation phase of acute inflammation. In our study, a high level of serum MMP3 at week 2 from baseline manifested as an effective predictor of PNR to IFX. Similarly, previous study had found that a high serum MMP3 concentration at week 14 was associated with a loss of response after 52 weeks of IFX treatment (16). Biancheri et al. showed that MMP3 was involved in cleaving anti-TNF agents, such as infliximab, adalimumab, and etanercept, in the colon mucosa of inflammatory bowel disease patients (17). This may explain the predictive function of MMP3 in PNR to IFX. However, whether MMP3 can predict a loss of response to other anti-TNF drugs has not been studied.

CCL2, namely monocyte chemotactic protein 1, is a CC-type chemokine that plays an important role in regulating migration and infiltration of monocytes, lymphocytes, or other immunocytes to inflammation sites in the pathogenesis of chronic inflammatory diseases such as CD (18–20). Previous studies have shown that the expression of CCL2 in intestinal epithelial cells, mucosa, circulating monocytes, or plasma was significantly increased in CD patients, especially those with active CD, compared to healthy individuals (21–23). Thus, CCL2 may be a pro-inflammatory factor in CD. Swedish patients with ulcerative colitis responding to infliximab therapy have been shown to express decreased levels of CCL2 in the serum at Week 2 (24). However, it is confusing that CD patients with PNR had lower serum CCL2 concentrations at week 2 after IFX treatment than responders in our study. A hypothesis that might explain this phenomenon is that the high serum concentration of CCL2 in primary responders is attributable to the decrease in CCR2 expression. A recent study showed that serum CCL2 was removed in a CCR2-dependent but G-protein independent manner, and when CCR2 expression decreased, serum CCL2 concentrations increased (25). Furthermore, anti-TNF non-responders with inflammatory bowel disease had upregulated CCR2 expression (26), which might cause lower serum CCL2 levels than those seen in the responders. Although this theoretical explanation makes sense, experimental validation is lacking. Therefore, it is attractive to research the change and function of the CCL2-CCR2 axis in CD patients before and after anti-TNF therapy for further studies.

CRP is one of the most common biomarkers used in clinical practice and is also useful for monitoring clinical or endoscopic activity in CD patients (27, 28). Post-injection CRP levels are a potential predictor for loss of response to anti-TNF therapy (29, 30). However, it remains controversial whether the baseline CRP level can predict loss of response to anti-TNF therapy in CD (31, 32). Our study found that serum CRP at week 2, but not baseline, was an independent predictor for PNR. However, CRP reflects the status of generic inflammation, not specific inflammatory pathways. In this study, we combined CRP with MMP3 and CCL2 levels, which are closely correlated with the pathogenesis of CD, and developed a well-founded PNR classifier.

Previous studies have demonstrated that the mechanisms underlying PNR are multifactorial. These mechanisms include disease characteristics, drug and treatment strategy-related factors, and inflammation and immune status of the patients (5). Disease duration, phenotype, and location may contribute to primary nonresponse. However, the role of patient-related characteristics was unclear because of controversial outcomes of different studies (5). Drug related factors includes through drug levels and the accumulation of anti-TNF antibodies. A recent study demonstrated that serum trough levels of IFX at week 2 could predict short-term clinical efficacy of IFX (33). Moreover, low IFX levels and high anti-TNF antibody levels to IFX at week 2 could predict PNR to IFX, with AUCs of 0.68 and 0.78, respectively (34). Nevertheless, the response status could be altered by changing the IFX dose or the combination of immunosuppressive drugs, and the effect of through drug levels and the anti-TNF antibodies could not count as primary mechanisms in the process of PNR. Emerging evidence suggest that primary nonresponse occurs in the patients with a disease course of non-TNF-driven inflammatory process. It has been thought that non-TNF-driven inflammatory processes were mainly involved in the mechanisms underlying PNR. Our research mainly focused on the non-TNF-driven inflammatory processes. We assumed that the changes of inflammatory states, which is presented by the alterations of the cytokines, chemokines and other inflammatory related factors, were significantly different between patients with TNF-driven inflammatory pathway and those were not. In the end, we explored MMP3 and CCL2 which were correlated with PNR.

There are some limitations to our study. From an analysis perspective, the reference cytokine subset we used as the discovery cohort to identify potential biomarkers is not a complete compendium of all of the immune related factors in the CD patients. Nevertheless, these potential factors have all been previously described in CD, which could compensate for the selection bias to some extent. In addition, the initial screening of cytokines in the discovery cohort was based on only 18 patients, which might be insubstantial to yield conclusive data. However, we screened potential biomarkers through analyses based not only on these 18 patients but also the patients in Cohort 1. we verified these potential cytokines selected from the discovery cohort in cohort 1 by univariate and multivariate analyses, which could reduce the incidence of false positives to a certain extent. Moreover, we did not detect IFX trough levels or anti-TNF antibody concentrations in the patients. These two indicators were thought to be able to predict loss of response of IFX (35, 36). However, this study mainly discussed the primary non-response of IFX, while trough serum concentrations of drug and anti-TNF antibodies are probably more relevant for the secondary loss of response (10). Another limitation is that all patients in our study are Chinese, which might make our results with limited generalisability.

In conclusion, our prospective and retrospective cohort study showed that CCL2 and MMP3 at week 2 play important roles in predicting PNR to IFX in CD patients. Furthermore, a PNR classifier was developed and proved effective in predicting PNR. However, further studies need to be performed on other patient populations to further substantiate our findings.

## Data Availability Statement

The raw data supporting the conclusions of this article will be made available by the authors, without undue reservation.

## Ethics Statement

This study was approved by the IEC for Clinical Research and Animal Trials of the First Affiliated Hospital of Sun Yat-sen University (No. 2019-383). Written informed consent to participate in this study was provided by the participants’ legal guardian/next of kin. Written informed consent was obtained from the individual(s), and minor(s)’ legal guardian/next of kin, for the publication of any potentially identifiable images or data included in this article.

## Author Contributions

All authors were responsible for the study concept and design. LL and RC: drafting of manuscript. LL, RC, YZ, and GZ analyzed the serum samples and interpretation of the data. RC and LL performed the statistical analysis. BC ZZ, MC, and SZ: critical revision of manuscript for important intellectual content. All authors contributed to the article and approved the submitted version.

## Funding

This project was supported by grants from the National Natural Science Foundation of China (#81630018, #81670498, #81870374, #82070538), Guangzhou Science and Technology Department (#202002030041), Guangdong Science and Technology (#2017A030306021, #2020A1515010249), China Postdoctoral Science Foundation (#2019M653228), and Science and Technology Innovation Young Talents of Guangdong Special Support Plan (#2016TQ03R296).

## Conflict of Interest

The authors declare that the research was conducted in the absence of any commercial or financial relationships that could be construed as a potential conflict of interest.
