# Supplementary material for: A Novel Model Based on Serum Biomarkers to Predict Primary Non-Response to Infliximab in Crohn’s Disease
Source: Front Immunol. 2021 Jul 22;12:646673. doi: 10.3389/fimmu.2021.646673 (PMC8339550; doi:10.3389/fimmu.2021.646673)
Supplement: Supplementary file 1 [file Table_1.docx]

**Supplement Table 1.** Baseline characteristics of Discovery cohort

|  | Discovery cohort (n=18) |
| --- | --- |
| Male | 13 (72.2) |
| Primary non-responders | 9 (50) |
| Age at 1^st^ IFX therapy (years) | 22.7 (18.5-26.8) |
| Body-mass index (kg/m²) | 16.3 (15.6-18.5) |
| Age at diagnosis (years) | 19.13 (16.62-22.56) |
| Disease duration (years) | 1.00 (0.40-5.63) |
| Disease location |  |
| L1 (ileal disease) | 2 (11.1) |
| L2 (colonic disease) | 1 (5.6) |
| L3 (ileocolonic disease) | 15 (83.3) |
| Presence of upper GI disease | 4 (22.2) |
| Disease behavior |  |
| B1 (non stricturing, non penetrating) | 13 (72.2) |
| B2 (stricturing) | 4 (22.2) |
| B3 (penetrating) | 1 (5.6) |
| Perianal disease | 6 (33.3) |
| Presence of extraintestinal manifestations | 2 (11.1) |
| Previous surgery | 4 (22.2) |
| History of smoking | 1 (5.6) |
| Concomitant Azathioprine | 10 (55.6) |
| CDAI score | 257 (228-279) |
| C-reactive protein (mg/L) | 19.2 (7.8-39.2) |
| Erythrocyte sedimentation rate (mm/h) | 69.5 (31.0-89.0) |
| Albumin (g/L) | 36.5 (32.9-37.9) |
| Haemoglobin (g/L) | 108 (96-128) |
| Platelet count (×10^9^/L) | 293 (251-429) |

Continuous variables and categorical variables are described as median (IQR) and n (%), respectively.

IQR: interquartile range; GI: gastrointestinal; CDAI: Crohn’s disease activity index
