# Supplementary material for: A Novel Model Based on Serum Biomarkers to Predict Primary Non-Response to Infliximab in Crohn’s Disease
Source: Front Immunol. 2021 Jul 22;12:646673. doi: 10.3389/fimmu.2021.646673 (PMC8339550; doi:10.3389/fimmu.2021.646673)
Supplement: Supplementary file 2 [file Table_2.docx]

**Supplement Table 2.** Baseline characteristics of primary non-response patients in cohort 1 and cohort 2

|  | Cohort 1 | Cohort 2 | P value |
| --- | --- | --- | --- |
| Primary non-responders | 12 | 45 | 0.925 |
| Failure of CDAI reduction | 10 (83.3) | 38 (84.4) |  |
| Therapy alteration | 2 (16.7) | 7 (15.6) |  |
| Male | 9 (75.0) | 32 (71.1) | 0.790 |
| Age at 1^st^ IFX therapy (years) | 24.38 (19.2-34.7) | 28.8 (22.6-36.5) | 0.318 |
| Body-mass index (kg/m²) | 17.7 (16.0-21.3) | 17.4 (15.8-18.8) | 0.570 |
| Age at diagnosis (years) | 22.6 (18.3-27.0) | 23.0 (18.3-29.3) | 0.512 |
| Disease duration (years) | 1.3 (0.8-8.4) | 4.6 (1.0-6.9) | 0.422 |
| Disease location |  |  | 0.175 |
| L1 (ileal disease) | 2 (16.7) | 11 (24.4) |  |
| L2 (colonic disease) | 0 (0) | 1 (2.2) |  |
| L3 (ileocolonic disease) | 10 (83.8) | 33 (73.3) |  |
| Presence of upper GI disease | 2 (16.7) | 9 (20.2) | 0.795 |
| Disease behavior |  |  | 0.344 |
| B1 (non stricturing, non penetrating) | 6 (50.0) | 13 (28.9) |  |
| B2 (stricturing) | 4 (33.3) | 25 (55.6) |  |
| B3 (penetrating) | 2 (16.7) | 7 (15.6) |  |
| Perianal disease | 4 (33.3) | 14 (31.1) | 0.833 |
| Presence of extraintestinal manifestations | 2 (16.7) | 11 (24.4) | 0.568 |
| Previous surgery | 2 (16.7) | 13 (28.9) | 0.393 |
| History of smoking | 0 (0) | 3 (6.7) | 0.358 |
| Concomitant Azathioprine | 8 (66.7) | 19 (42.2) | 0.132 |
| CDAI score | 268 (194-296) | 232 (198-245) | 0.352 |
| C-reactive protein (mg/L) | 33.0 (19.3-58.6) | 19.4 (2.3-35.0) | 0.069 |
| Erythrocyte sedimentation rate (mm/h) | 69.0 (47.5-85.8) | 38.0 (19.5-60.5) | 0.015 |
| Albumin (g/L) | 34.0 (30.8-36.5) | 35.4 (31.2-38.2) | 0.318 |
| Haemoglobin (g/L) | 113 (100-127) | 114 (95-135) | 0.807 |
| Platelet count (×10^9^/L) | 364 (301-442) | 343 (267-459) | 0.493 |

Continuous variables and categorical variables are described as median (IQR) and n (%), respectively.

IQR: interquartile range; GI: gastrointestinal; CDAI: Crohn’s disease activity index
