# Supplementary material for: A Novel Model Based on Serum Biomarkers to Predict Primary Non-Response to Infliximab in Crohn’s Disease
Source: Front Immunol. 2021 Jul 22;12:646673. doi: 10.3389/fimmu.2021.646673 (PMC8339550; doi:10.3389/fimmu.2021.646673)
Supplement: Supplementary file 3 [file Table_3.docx]

**Supplement Table 3.** Comparison of CRP, ESR, SESCD at week 14 between primary responders and primary non-responders.

|  | Cohort 1 | | P value | Cohort 2 | | P value |
| --- | --- | --- | --- | --- | --- | --- |
|  | Primary responders | Primary non-responders |  | Primary responders | Primary non-responders |  |
| CRP (mg/L) | 1.2 (0.8-6.5) | 13.0 (8.1-55.7) | <0.001 | 1.9 (0.8-5.5) | 10.0 (5.8-28.7) | <0.001 |
| ESR (mm/h) | 9.5 (5.0-24.0) | 54.0 (35.0-64.8) | <0.001 | 14.0 (5.5-29.5) | 11.0 (22.0-65.0) | <0.001 |
| Number of endoscopy procedures | 40 (83.3) | 11 (91.7) |  | 79 (56.9) | 33 (73.3) |  |
| SESCD | 6 (4-10) | 14 (12-24) | <0.001 | 4 (2-7) | 12 (7-21) | <0.001 |

Continuous variables and categorical variables are described as median (IQR) and n (%), respectively.

IQR: interquartile range; CRP: c-reactive protein; ESR: erythrocyte sedimentation rate; SESCD: Simple Endoscopic Score for Crohn’s Disease.
