# Supplementary material for: A Novel Model Based on Serum Biomarkers to Predict Primary Non-Response to Infliximab in Crohn’s Disease
Source: Front Immunol. 2021 Jul 22;12:646673. doi: 10.3389/fimmu.2021.646673 (PMC8339550; doi:10.3389/fimmu.2021.646673)
Supplement: Supplementary file 4 [file Table_4.docx]

**Supplement Table 4.** Median and interquartile ranges of serum cytokines in primary responders and primary non-responders to infliximab in discovery cohort.

|  | Primary responders  (n=9) | Primary non-responders (n=9) | P value | Adjusted p value* |
| --- | --- | --- | --- | --- |
| **At baseline** |  |  |  |  |
| CCL2 pg/ml | 271.7 (258.4-412.5) | 262.7 (250.3-358.1) | 0.561 | 1.000 |
| CCL4 pg/ml | 674.7 (646.9-835.7) | 690.2 (634.8-897.1) | 0.931 | 1.000 |
| CCL11 pg/ml | 207.7 (177.4-274.7) | 239.8 (221.7-316.1) | 0.190 | 1.000 |
| CCL20 pg/ml | 76.7 (63.1-160.4) | 93.1 (67.8-181) | 0.730 | 1.000 |
| CCL25 pg/ml | 244.9 (182.6-414.9) | 232.3 (167.2-310.3) | 0.622 | 1.000 |
| CCL26 pg/ml | 44.8 (30.5-58.3) | 34.3 (29.4-45.2) | 0.308 | 1.000 |
| CX3CL1 pg/ml | 1930 (1452-2288) | 2062 (1690.5-2342.5) | 0.623 | 1.000 |
| CXCL8 pg/ml | 19.8 (17.6-70.1) | 18.6 (16.6-55.4) | 0.666 | 1.000 |
| CXCL11 pg/ml | 199.8 (155.9-236.2) | 196.7 (153.8-233.7) | 0.780 | 1.000 |
| ADAMTS13 pg/ml | 9.13*10^5^ (8.45*10^5^-13.2*10^5^) | 9.22*10^5^ (5.35*10^5^-14.0*10^5^) | 0.730 | 1.000 |
| Adiponectin pg/ml | 8.74*10^5^ (8.42*10^5^-9.26*10^5^) | 9.18*10^5^ (8.64*10^5^-9.58*10^5^) | 0.297 | 1.000 |
| alpha 2-Macroglobulin pg/ml | 5.33*10^5^ (5.14*10^5^-5.52*10^5^) | 5.14*10^5^ (4.74*10^5^-5.28*10^5^) | 0.109 | 1.000 |
| IGFBP-1 pg/ml | 56468 (17664.5-108794.5) | 21462 (14163-107899) | 0.605 | 1.000 |
| MMP-3 pg/ml | 15580 (13518.5-17703.5) | 22545 (15852.5-36265) | 0.050 | 1.000 |
| TNF-alpha pg/ml | 19.1 (16-28.2) | 18.8 (16.6-20.3) | 0.713 | 1.000 |
| IL-1 beta pg/ml | 18.9 (13.8-32.8) | 22.5 (19.2-28.4) | 0.489 | 1.000 |
| IL-2 pg/ml | 385.5 (341.8-616.7) | 379.4 (304.2-650.2) | 0.863 | 1.000 |
| IL-4 pg/ml | 133.8 (104.6-163.9) | 159.3 (123.1-254.9) | 0.222 | 1.000 |
| IL-6 pg/ml | 10 (7.1-22.6) | 12.9 (11.1-18.6) | 0.387 | 1.000 |
| IL-7 pg/ml | 26 (22.2-28.9) | 18.2 (15.6-31.7) | 0.297 | 1.000 |
| IL-10 pg/ml | 8.4 (7.6-9.6) | 8.2 (6.7-12.2) | 0.747 | 1.000 |
| IL-11 pg/ml | 2462 (2302.5-2860.5) | 2651 (2483-3006) | 0.308 | 1.000 |
| IL-12 p70 pg/ml | 87.5 (74.8-106.1) | 88.5 (56.2-111) | 0.681 | 1.000 |
| IL-13 pg/ml | 1376 (994.5-1472.5) | 1610 (1226-2543.5) | 0.142 | 1.000 |
| IL-17 pg/ml | 22.6 (13.4-52.7) | 28.4 (20.2-35.9) | 0.502 | 1.000 |
| IL-18 pg/ml | 604.8 (572.1-774.1) | 692.9 (650.4-1417.8) | 0.197 | 1.000 |
| IL-23 pg/ml | 744.2 (617.2-1059.4) | 905 (800.8-978.4) | 0.249 | 1.000 |
| IL-27 pg/ml | 1487 (1270-1664.5) | 1510 (1224.5-1988.5) | 0.845 | 1.000 |
| IL-28A pg/ml | 384.1 (238.5-500.5) | 319.4 (246.6-610.9) | 0.748 | 1.000 |
| IL-28B pg/ml | 790.2 (664.7-1047) | 1011 (800.9-1199.5) | 0.136 | 1.000 |
| IL-31 pg/ml | 146.6 (115.3-197.9) | 110.3 (95.7-167.2) | 0.190 | 1.000 |
| IL-33 pg/ml | 10.5 (9.3-21.1) | 14.6 (11.8-27.7) | 0.423 | 1.000 |
| IL-34 pg/ml | 114.3 (83-153.6) | 118.9 (104.1-132.5) | 0.561 | 1.000 |
| IL-36 beta pg/ml | 18.8 (14.1-24.7) | 17.6 (13.6-25.9) | 0.931 | 1.000 |
| **At week 2** |  |  |  | 1.000 |
| CCL2 pg/ml | 314.5 (245.1-326.5) | 230.7 (201.3-265.9) | 0.013 | 1.000 |
| CCL4 pg/ml | 643.1 (553.6-719.9) | 697.8 (601.7-930.9) | 0.307 | 1.000 |
| CCL11 pg/ml | 203.4 (184.7-274.7) | 214.8 (178.8-256.1) | 0.863 | 1.000 |
| CCL20 pg/ml | 45.5 (37.2-67.9) | 62.5 (43.5-83.9) | 0.436 | 1.000 |
| CCL25 pg/ml | 212 (205.5-241.2) | 199.8 (163.2-225.8) | 0.222 | 1.000 |
| CCL26 pg/ml | 46 (31.3-59.6) | 38.8 (28.1-43.6) | 0.222 | 1.000 |
| CX3CL1 pg/ml | 2147 (1881.5-2570) | 2003 (1703-2218.5) | 0.214 | 1.000 |
| CXCL8 pg/ml | 13.9 (13.2-28.3) | 16.1 (15.3-42.8) | 0.248 | 1.000 |
| CXCL11 pg/ml | 126 (110.8-214) | 178 (138.3-190.5) | 0.730 | 1.000 |
| ADAMTS13 pg/ml | 10.44*10^5^ (6.98*10^5^-13.56*10^5^) | 9.51*10^5^ (5.62*10^5^-1.37*10^5^) | 0.489 | 1.000 |
| Adiponectin pg/ml | 9.08*10^5^ (8.35*10^5^-9.66*10^5^) | 9.45*10^5^ (9.05*10^5^-9.57*10^5^) | 0.666 | 1.000 |
| alpha 2-Macroglobulin pg/ml | 5.94*10^5^ (5.30*10^5^-6.68*10^5^) | 5.43*10^5^ (5.21*10^5^9-5.70*10^5^) | 0.190 | 1.000 |
| IGFBP-1 pg/ml | 12129 (10253-24287) | 18481 (7096-35256) | 1.000 | 1.000 |
| MMP-3 pg/ml | 8656 (6294.5-11055.5) | 13658 (11423.5-27803) | 0.008 | 0.816 |
| TNF-alpha pg/ml | 18.1 (15.6-30.2) | 23.5 (16.3-38.4) | 0.503 | 1.000 |
| IL-1 beta pg/ml | 16.3 (13.5-30.1) | 20.8 (15.4-44) | 0.449 | 1.000 |
| IL-2 pg/ml | 450.7 (376.3-712.9) | 409.7 (322.4-521.6) | 0.387 | 1.000 |
| IL-4 pg/ml | 138.5 (109-198.7) | 148.6 (119.2-217.8) | 0.562 | 1.000 |
| IL-6 pg/ml | 9.9 (4.6-11.2) | 9.4 (7-17.6) | 0.374 | 1.000 |
| IL-7 pg/ml | 16.7 (14.4-21.3) | 17 (12.1-28.4) | 0.982 | 1.000 |
| IL-10 pg/ml | 9.9 (7-11.2) | 9.1 (7.9-11.8) | 0.880 | 1.000 |
| IL-11 pg/ml | 1993 (1804.5-3414.5) | 2651 (2148-3182) | 0.154 | 1.000 |
| IL-12 p70 pg/ml | 132 (78.8-141.1) | 97 (66.6-135.4) | 0.589 | 1.000 |
| IL-13 pg/ml | 1344 (1132-2019.5) | 1344 (1244-2395) | 0.812 | 1.000 |
| IL-17 pg/ml | 21.6 (12.5-48.8) | 25.5 (20.9-54.9) | 0.589 | 1.000 |
| IL-18 pg/ml | 662.8 (578.3-776.8) | 647.7 (544-1724) | 0.863 | 1.000 |
| IL-23 pg/ml | 776.6 (670.7-1048.5) | 897.1 (839.1-1151.5) | 0.182 | 1.000 |
| IL-27 pg/ml | 1327 (1172.5-1653) | 1442 (1127-1818.5) | 0.949 | 1.000 |
| IL-28A pg/ml | 319.4 (287-551.7) | 384.1 (198.5-540.1) | 0.915 | 1.000 |
| IL-28B pg/ml | 944.2 (637.2-978) | 1086 (707.6-1193.5) | 0.142 | 1.000 |
| IL-31 pg/ml | 164.1 (108.5-197.9) | 133.6 (80.9-162.6) | 0.190 | 1.000 |
| IL-33 pg/ml | 9.9 (8.2-21.4) | 12.4 (9.8-19.9) | 0.650 | 1.000 |
| IL-34 pg/ml | 131.4 (86.3-160.4) | 116.6 (96.8-142.8) | 0.621 | 1.000 |
| IL-36 beta pg/ml | 17.2 (10.5-36.2) | 14.9 (11.3-20) | 0.780 | 1.000 |
| **Change from baseline to week 2** |  |  |  | 1.000 |
| CCL2 pg/ml | -30.4 (-97.2-48.7) | -42.5 (-120.1-12.2) | 0.605 | 1.000 |
| CCL4 pg/ml | -73.3 (-134-53.5) | -32.5 (-89.6-24.9) | 0.489 | 1.000 |
| CCL11 pg/ml | 2.6 (-44.5-34.1) | -16.7 (-71.5--6.2) | 0.258 | 1.000 |
| CCL20 pg/ml | -38.3 (-111.9-20.1) | -57.6 (-102.8-8.8) | 0.931 | 1.000 |
| CCL25 pg/ml | -34.7 (-207.7-33) | -58.8 (-106.3-30.3) | 0.863 | 1.000 |
| CCL26 pg/ml | 1.5 (0.6-5.3) | -0.5 (-2.6-3) | 0.119 | 1.000 |
| CX3CL1 pg/ml | 248 (-48-555.5) | 96 (-515-199) | 0.340 | 1.000 |
| CXCL8 pg/ml | -5.5 (-14.7--0.5) | -3.1 (-9.1--0.7) | 0.730 | 1.000 |
| CXCL11 pg/ml | -44 (-64.8--29.7) | -31 (-57.1--5.2) | 0.387 | 1.000 |
| ADAMTS13 pg/ml | 28442 (-134207.5-100870) | -12909 (-197248.5-121306.5) | 0.863 | 1.000 |
| Adiponectin pg/ml | 26467 (-4268-44891.5) | 701 (-20079-58331.5) | 0.863 | 1.000 |
| alpha 2-Macroglobulin pg/ml | 79954 (-21540.5-116601.5) | 18945 (8640-64751.5) | 0.489 | 1.000 |
| IGFBP-1 pg/ml | -25200 (-92124.5-300) | -14585 (-56386--3473) | 0.666 | 1.000 |
| MMP-3 pg/ml | -6273 (-9663--1683.5) | -5730 (-13437--1213.5) | 1.000 | 1.000 |
| TNF-alpha pg/ml | 0.3 (-2.2-4.7) | 3.1 (0.5-17.3) | 0.297 | 1.000 |
| IL-1 beta pg/ml | -1.3 (-3.9-0.8) | 0.7 (-9-16) | 0.666 | 1.000 |
| IL-2 pg/ml | 45.4 (-18.9-112.3) | 23.4 (-152.7-93.8) | 0.387 | 1.000 |
| IL-4 pg/ml | 6.4 (-11.7-29.2) | -5.8 (-61.1-7.8) | 0.297 | 1.000 |
| IL-6 pg/ml | -3.1 (-12-0.1) | -3.3 (-8.6-4.3) | 0.931 | 1.000 |
| IL-7 pg/ml | -7.2 (-13.9--3.2) | -3 (-4.7--1) | 0.050 | 1.000 |
| IL-10 pg/ml | 0.6 (-1.6-1.7) | 0.3 (-1.4-1.4) | 0.863 | 1.000 |
| IL-11 pg/ml | -430 (-519.5-510) | -275 (-472-394) | 0.666 | 1.000 |
| IL-12 p70 pg/ml | 15.7 (-20.5-55.6) | 0 (-7.9-49.1) | 0.982 | 1.000 |
| IL-13 pg/ml | 43 (-330-743) | -154 (-339--30.5) | 0.297 | 1.000 |
| IL-17 pg/ml | 0.9 (-3.4-5.3) | 0 (-2-8.5) | 0.952 | 1.000 |
| IL-18 pg/ml | 53.6 (-81.8-93.5) | 11.4 (-175.5-407) | 1.000 | 1.000 |
| IL-23 pg/ml | 64.7 (-46.7-103.5) | 61 (-77.9-189.2) | 0.796 | 1.000 |
| IL-27 pg/ml | -126 (-183-188.5) | -103 (-364.5-159.5) | 0.560 | 1.000 |
| IL-28A pg/ml | 56.6 (-36.4-107.8) | -39.2 (-87.4-56.6) | 0.222 | 1.000 |
| IL-28B pg/ml | -17.3 (-50.2-52.8) | 39.8 (-149.1-131.3) | 0.561 | 1.000 |
| IL-31 pg/ml | 9.7 (-21.2-35.8) | 3.2 (-24.4-21.4) | 0.666 | 1.000 |
| IL-33 pg/ml | -0.4 (-3.1-2) | -1.1 (-7.5-2.5) | 0.531 | 1.000 |
| IL-34 pg/ml | 4.3 (-7.4-39.3) | 11.4 (-23.2-25.5) | 0.546 | 1.000 |
| IL-36 beta pg/ml | -2.3 (-3.6-12.7) | -4.3 (-6.1-0.6) | 0.190 | 1.000 |

*The p value was adjusted by False Discovery Rate (Benjaminiand-Hochberg method).

CCL8 was undetectable among more than half of the patients, so the levels of serum CCL8 were not showed.

Abbreviation: CCL: C-C motif ligand; MMP: matrix metalloproteinase; IL: interleukin; TNF: tumour necrosis factor.
