# Supplementary material for: A Novel Model Based on Serum Biomarkers to Predict Primary Non-Response to Infliximab in Crohn’s Disease
Source: Front Immunol. 2021 Jul 22;12:646673. doi: 10.3389/fimmu.2021.646673 (PMC8339550; doi:10.3389/fimmu.2021.646673)
Supplement: Supplementary file 5 [file Table_5.docx]

**Supplement Table 5.** Univariate association analyses of clinical characteristics and serological markers for primary non-response in cohort 2

|  | Primary responders (n=137) | Primary non-responders (n=45) | P value |
| --- | --- | --- | --- |
| **Clinical characteristics** |  |  |  |
| Male | 96 (70.1) | 32 (71.1) | 0.895 |
| Age at 1^st^ IFX therapy (years) | 23.6 (18.3-32.6) | 28.8 (22.6-36.5) | 0.044 |
| Body-mass index (kg/m²) | 17.8 (16.2-19.4) | 17.4 (15.8-18.8) | 0.391 |
| Age at diagnosis (years) | 20.0 (16.5-29.0) | 23.0 (18.3-29.3) | 0.165 |
| Disease duration (years) | 1.2 (0.6-4.0) | 4.6 (1.0-6.9) | 0.002 |
| Disease location |  |  | 0.196 |
| L1 (ileal disease) | 19 (13.9) | 11 (24.4) |  |
| L2 (colonic disease) | 7 (5.1) | 1 (2.2) |  |
| L3 (ileocolonic disease) | 111 (81.0) | 33 (73.3) |  |
| Presence of upper GI disease | 33 (24.1) | 9 (20.0) | 0.572 |
| Disease behavior |  |  | 0.001 |
| B1 (non stricturing, non penetrating) | 82 (59.9) | 13 (28.9) |  |
| B2 (structuring) | 37 (27.0) | 25 (55.6) |  |
| B3 (penetrating) | 18 (13.1) | 7 (15.6) |  |
| Perianal disease | 56 (40.9) | 14 (31.1) | 0.243 |
| Presence of extraintestinal manifestations | 23 (16.8) | 11 (24.4) | 0.253 |
| Previous surgery | 27 (19.7) | 13 (28.9) | 0.197 |
| History of smoking | 12 (8.8) | 3 (6.7) | 0.766 |
| Concomitant Azathioprine | 60 (43.8) | 19 (42.2) | 0.853 |
| CDAI score at baseline | 237 (199-286) | 232 (198-245) | 0.473 |
| **Level of serological markers at baseline** |  |  |  |
| CRP (mg/L) | 22.7 (8.9-41.3) | 19.4 (2.3-35.0) | 0.144 |
| Erythrocyte sedimentation rate (mm/h) | 45.0 (25.0-75.5) | 38.0 (19.5-60.5) | 0.075 |
| Albumin (g/L) | 35.0 (31.1-39.0) | 35.4 (31.2-38.2) | 0.927 |
| Haemoglobin (g/L) | 112 (96-125) | 114 (95-135) | 0.393 |
| White blood cell (×10^9^/L) | 7.4 (6.0-9.1) | 6.9 (5.2-8.6) | 0.310 |
| Neutrophil (×10^9^/L) | 4.9 (3.8-6.1) | 4.9 (3.6-5.9) | 0.425 |
| Neutrophil percentage (%) | 67.6 (60.9-73.4) | 67.9 (62.8-74.0) | 0.605 |
| Lymphocytes (×10^9^/L) | 1.4 (1.1-1.8) | 1.2 (0.9-1.9) | 0.120 |
| Lymphocyte percentage (%) | 19.6 (14.9-24.6) | 18.6 (15.3-26.3) | 0.911 |
| Platelet count (×10^9^/L) | 350 (288-435) | 343 (267-459) | 0.685 |
| PLR | 258 (188-330) | 249 (196-356) | 0.746 |
| NLR | 3.4 (2.5-4.9) | 3.7 (2.3-4.9) | 0.766 |
| **Level of serological markers at weeks 2** |  |  |  |
| CRP (mg/L) | 1.5 (0.8-3.6) | 4.0 (1.0-8.7) | <0.001 |
| Erythrocyte sedimentation rate (mm/h) | 20.0 (10.0-40.0) | 18 (11-39) | 0.953 |
| Albumin (g/L) | 39.0 (36.0-42.5) | 37.0 (33.5-41.2) | 0.070 |
| Haemoglobin (g/L) | 118 (103-130) | 116 (95-133) | 0.502 |
| White blood cell (×10^9^/L) | 5.7 (4.6-6.9) | 6.0 (4.6-7.6) | 0.302 |
| Neutrophil (×10^9^/L) | 3.1 (2.5-4.4) | 4.0 (2.6-5.1) | 0.145 |
| Neutrophil percentage (%) | 58.6 (51.8-64.1) | 63.6 (55.8-71.7) | 0.010 |
| Lymphocytes (×10^9^/L) | 1.6 (1.3-2.0) | 1.4 (1.1-1.9) | 0.042 |
| Lymphocyte percentage (%) | 30.6 (24.5-35.1) | 24.9 (17.8-32.2) | 0.007 |
| Platelet count as baseline (×10^9^/L) | 292 (235-349) | 302 (245-361) | 0.584 |
| PLR | 180 (142-230) | 217 (152-291) | 0.028 |
| NLR | 2.0 (1.5-2.6) | 2.5 (1.7-3.8) | 0.008 |
| CCL2 (pg/ml) | 98.9 (80.0-120.5) | 72.0 (55.7-88.0) | <0.001 |
| MMP3 (ng/ml) | 12.7 (8.1-17.4) | 24.7 (16.0-33.6) | <0.001 |

Abbreviation: CRP: C-reactive protein; PLR: platelet-to-lymphocyte ratio; NLR: neutrophil-to-lymphocyte ratio; CCL: C-C motif ligand; MMP: matrix metalloproteinase.
