# Supplementary material for: A Novel Model Based on Serum Biomarkers to Predict Primary Non-Response to Infliximab in Crohn’s Disease
Source: Front Immunol. 2021 Jul 22;12:646673. doi: 10.3389/fimmu.2021.646673 (PMC8339550; doi:10.3389/fimmu.2021.646673)
Supplement: Supplementary file 6 [file Table_6.docx]

**Supplement Table 6.** Multivariate analyses for the variables associated with primary non-response in patients determined response status by CDAI in cohort 2

|  | Odds Ratio (95% CI) | P value |
| --- | --- | --- |
| **Clinical characteristics** |  |  |
| Age at 1^st^ IFX therapy (years) | 1.008 (0.941-1.079) | 0.821 |
| Disease duration (years) | 1.017 (0.875-1.181) | 0.826 |
| CD behaviour | 1.293 (0.598-2.797 | 0.541 |
| **Level of serological markers at weeks 2** |  |  |
| Neutrophil percentage (%) | 1.014 (0.893-1.151) | 0.833 |
| Lymphocytes (×10^9^/L) | 2.492 (0.608-10.200) | 0.204 |
| Lymphocyte percentage (%) | 0.928 (0.787-1.095) | 0.377 |
| PLR | 1.006 (0.997-1.015) | 0.188 |
| NLR | 0.787 (0.215-2.879) | 0.717 |
| CRP (mg/L) | 1.097 (1.016-1.185) | 0.018 |
| CCL2 (pg/ml) | 0.937 (0.912-0.984) | <0.001 |
| MMP3 (ng/ml) | 1.097 (1.050-1.146) | <0.001 |

Abbreviation: CRP: C-reactive protein; PLR: platelet-to-lymphocyte ratio; NLR: neutrophil-to-lymphocyte ratio; CCL: C-C motif ligand; MMP: matrix metalloproteinase.
