# Supplementary material for: A Novel Model Based on Serum Biomarkers to Predict Primary Non-Response to Infliximab in Crohn’s Disease
Source: Front Immunol. 2021 Jul 22;12:646673. doi: 10.3389/fimmu.2021.646673 (PMC8339550; doi:10.3389/fimmu.2021.646673)
Supplement: Supplementary file 7 [file Table_7.docx]

**Supplement Table 7.** The odds ratio (OR) of CRR, MMP3, CCL2 and PNR classifier in predicting primary non-response to IFX.

|  | Unadjusted  OR (95%CI) | Unadjusted  P value | Adjusted  OR (95%CI) * | Adjusted  P value |
| --- | --- | --- | --- | --- |
| CRP >3.79 mg/L | 7.118 (2.439-20.773) | <0.001 | 6.314 (1.956-20.377) | 0.002 |
| MMP3 > 19.15pg/ml | 6.998 (2.263-21.635) | 0.001 | 8.478 (2.307-31.160) | 0.001 |
| CLL2 < 84.84pg/ml | 3.716 (1.188-11.626) | 0.024 | 3.490 (1.026-11.866) | 0.045 |
| PNR classifier >0.331 | 7.728 (2.564-23.292) | <0.001 | 6.467 (1.928-21.687) | 0.002 |

*The OR (95% CI) was adjusted by clinical characteristics (age at 1^st^ IFX therapy, disease duration, CD behaviour) and concentration of serological biomarkers at week 2 (neutrophil percentage, lymphocytes, lymphocyte percentage, platelet-to-lymphocyte ratio and neutrophil-to-lymphocyte ratio.

Abbreviation: CRP: C-reactive protein; CCL: C-C motif ligand; MMP: matrix metalloproteinase; PNR: primary non-response.
